# Atypical Respiratory Presentation of Pediatric Achalasia: A Case Report

**DOI:** 10.7759/cureus.105109

**Published:** 2026-03-12

**Authors:** Jeremias Munoz, Amber McClain, Jacqueline Larson, Meghana Reddy

**Affiliations:** 1 Pediatrics, University of South Florida Morsani College of Medicine, Tampa, USA; 2 Pediatric Gastroenterology, University of South Florida Morsani College of Medicine, Tampa, USA; 3 Internal Medicine-Pediatrics, University of South Florida Morsani College of Medicine, Tampa, USA

**Keywords:** aspiration pneumonia, atypical presentation, esophageal achalasia, esophageal myotomy, pediatric achalasia, respiratory

## Abstract

Esophageal achalasia is a rare smooth muscle disorder characterized by the absence of peristalsis and impaired relaxation of the lower esophageal sphincter (LES), ultimately leading to obstruction and impaired bolus transit. Pediatric achalasia is especially uncommon and can present with diagnostic delay due to clinical overlap with more common gastrointestinal and pulmonary conditions. We report the case of a previously healthy 13-year-old girl who presented with a five-month history of persistent nocturnal cough, fevers, and progressive weight loss, repeatedly diagnosed and treated as bronchospasm and community-acquired pneumonia without improvement. Over time, she developed non-bloody, non-bilious emesis, decreased oral intake and tolerance, and dysphagia localized to the mid-chest, raising concern for obstructive esophageal pathology. Computed tomography (CT) of the chest revealed a markedly dilated, food- and fluid-filled esophagus with abrupt tapering at the gastroesophageal junction (GEJ), suggestive of achalasia. Diagnosis was confirmed with a timed barium esophagram and upper endoscopy with functional lumen imaging probe (FLIP). The patient underwent therapeutic botulinum toxin injection, with rapid symptom improvement, and nutritional rehabilitation, followed by surgical correction with thoracoscopic esophagomyotomy. This case highlights the potential for respiratory-dominant atypical presentations of achalasia in pediatric patients and the importance of early diagnosis and intervention to prevent long-term complications, including recurrent aspiration and impact on growth and development secondary to nutritional deficits.

## Introduction

Achalasia is a rare motility disorder of the esophagus characterized by impaired relaxation of the lower esophageal sphincter (LES) and ineffective peristalsis [1,2]. The condition arises from selective degeneration of inhibitory neurons in the myenteric plexus, leading to unopposed excitatory signaling and sustained LES contraction [3]. Although primarily recognized in adults, pediatric achalasia accounts for less than 5% of total cases and carries an estimated annual incidence of 0.10 cases per 100,000 person-years worldwide [1,2]. In children, diagnostic delays may occur due to symptom overlap with gastrointestinal conditions, as well as the latency between disease onset, symptom presentation, and seeking medical evaluation, resulting in nutritional deterioration [3].

In practice, achalasia most commonly manifests as dysphagia to both solids and liquids, and regurgitation of undigested food, as well as chest pain and variable weight loss [4-6]. Dysphagia is typically insidious and initially intermittent, progressing to involve difficulty with most food consistencies, as is characteristic of esophageal motility dysfunction [4,6]. Regurgitation may occur within minutes of eating and typically involves non-acidic food particulate; however, it can also lead to esophageal acidification through the fermentation of retained food, contributing to heartburn symptoms in up to 75% of patients [6]. The degree of regurgitation follows disease progression and, with sufficient severity, can less commonly result in bronchitis and recurrent pneumonia [3]. Mechanistically, such infections are the consequence of esophageal contents entering the airway during episodic regurgitation, introducing gastrointestinal bacterial flora in tandem with chemical insult from acidic gastric contents, providing a nidus for lower respiratory tract infections.

Although various modalities can support the diagnosis, definitive evidence of achalasia is obtained through high-resolution manometry (HRM), the current gold standard, which demonstrates high pressures at the LES and abnormal peristaltic action [3-6]. Upper endoscopy is essential for ruling out pseudoachalasia, which encompasses structural or alternative processes that produce analogous HRM patterns to achalasia, including distal esophageal, proximal gastric, and even small cell lung cancers [3,5,7]. A functional lumen imaging probe (FLIP) may provide adjuvant evaluation in equivocal cases by assessing the distensibility of the esophagus as a distensibility index and measuring luminal diameter within a 3D geometric representation, which is particularly useful in identifying candidates likely to benefit from intervention [3,4,6]. Timed barium esophagram and computed tomography (CT) of the chest serve as complementary diagnostic studies, with barium swallow studies often revealing a dilated esophagus with a narrowed gastroesophageal junction (GEJ), the "bird's beak" sign, and delayed contrast clearance, and chest CT demonstrating a dilated esophagus with food debris [7].

We present the case of a previously healthy adolescent girl with an atypical achalasia presentation of refractory respiratory symptoms preceding overt gastrointestinal manifestations.

## Case presentation

A previously healthy 13-year-old girl presented to the pediatric emergency department (ED) of a tertiary, academic medical center for persistent cough, fevers, and shortness of breath. Her symptoms developed over five months, initially presenting with fever, chills, and fatigue. She also experienced a predominantly nighttime cough described as copiously productive, to the extent that it caused nasal congestion and overflow while supine, necessitating nighttime awakening and sputum expulsion. Approximately two months prior to presentation, the patient was prescribed a course of amoxicillin by her primary care provider, but despite antibiotic therapy, her symptoms persisted. The patient had two additional ED visits for fevers and respiratory symptoms, where she was diagnosed with presumed pneumonia and bronchospasm, and discharged with albuterol, dexamethasone, azithromycin, and additional courses of amoxicillin, all of which provided no relief or resolution.

In addition to her respiratory concerns, the patient began experiencing episodes of non-bilious, non-bloody emesis, food intolerance, and epigastric abdominal pain one month prior to presentation. She described food, primarily solids, as feeling "stuck" in her mid-chest, although she denied difficulties with swallowing. An unintentional 20-pound weight loss was also noted across the five-month period. The family history was non-contributory, including no familial rheumatologic disease. There was no recent travel outside the United States, and she denied any additional symptoms of night sweats, myalgias, diarrhea, constipation, rashes, or joint pains. The patient was admitted for further evaluation and inpatient management, with consults including pediatric gastroenterology, infectious disease, and pulmonology.

On physical examination, the patient was thin, ill-appearing, tachycardic with a heart rate in the 130s, and tachypneic with an oxygen saturation of 90%, with a body mass index (BMI) of 15.04 kg/m^2^ (third percentile). Pertinent examination findings included rhinorrhea, mild wheezing, and crackles throughout the lung fields, as well as diffuse abdominal tenderness, most prominent in the left upper quadrant. The remainder of the physical examination was unremarkable.

**Figure 1:**
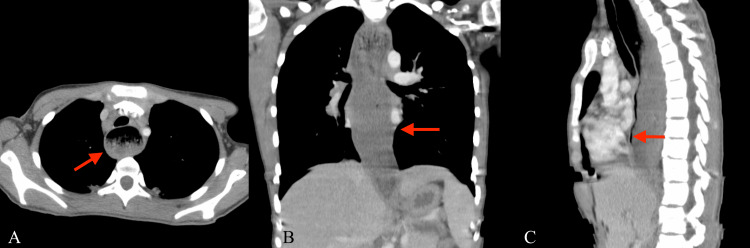
Chest CT with contrast in axial (A), coronal (B), and sagittal (C) views, demonstrating significant distention of the esophagus with fluid and food particulate throughout its course (red arrows) with abrupt tapering at the gastroesophageal junction CT: computed tomography

**Figure 2:**
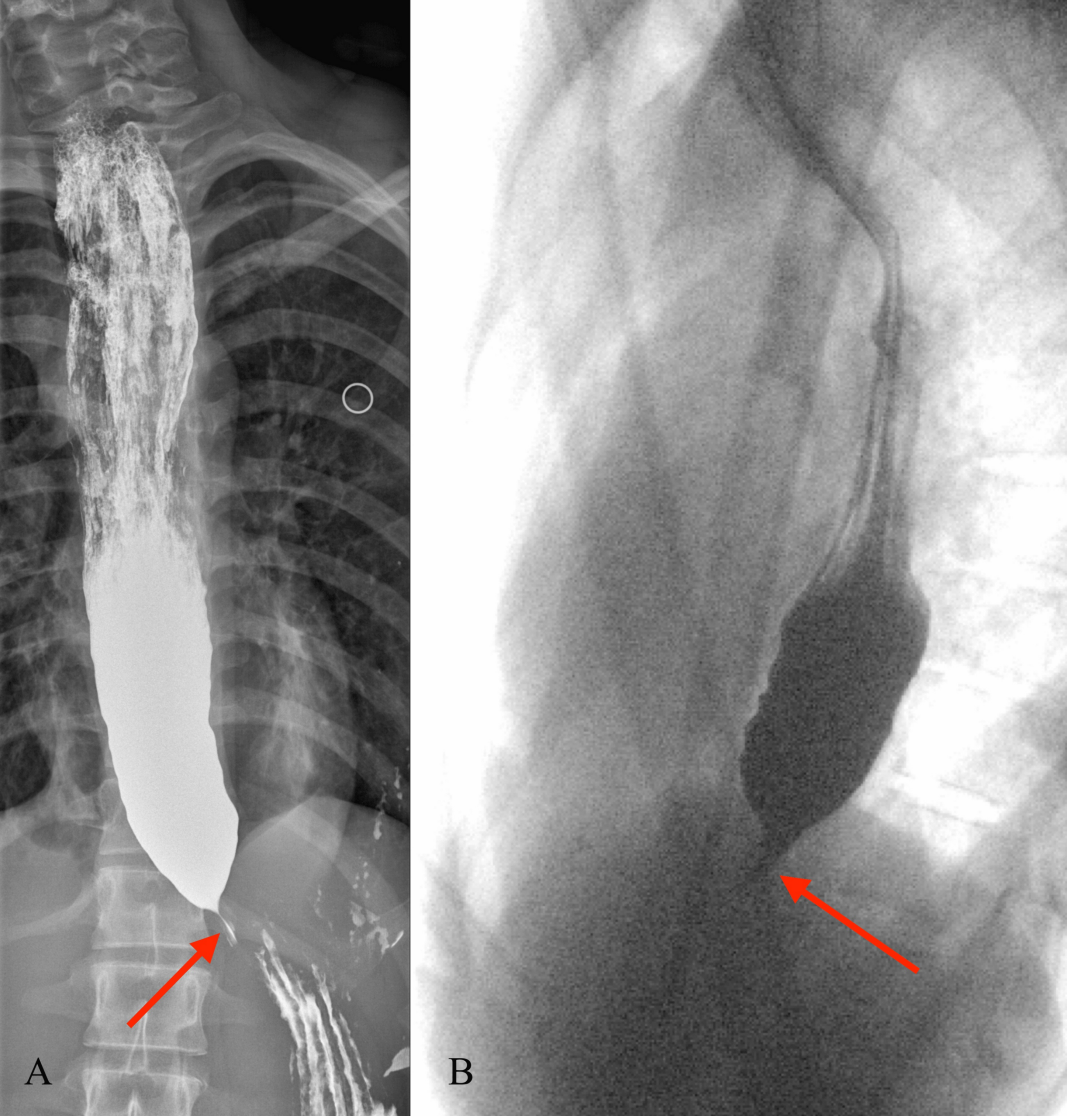
Esophagram results in axial (A) and sagittal (B) views, showing significant esophageal dilation and narrowing at the gastroesophageal junction with a classic "bird's beak" appearance (red arrows)

Laboratory workup (Table 1) was significant for leukocytosis of 20.10 × 10^3^/uL (normal: 4.6-10.2 × 10^3^/uL), hemoglobin of 10.9 g/dL (normal: 12.2-16.2 g/dL), platelets of 460 × 10^3^/uL (normal: 142-424 × 10^3^/uL), and elevated erythrocyte sedimentation rate (ESR) and C-reactive protein (CRP) of 82 mm/hour (normal: 0-20 mm/hour) and 5.98 mg/dL (normal: 0-0.5 mg/dL), respectively. Additional infectious workup and rheumatologic markers (Table 1) were predominantly negative, except for positive results for respiratory syncytial virus (RSV) and anti-histone antibodies. The following additional tests (Table 1) were also unremarkable: Epstein-Barr virus (EBV), cytomegalovirus (CMV), quantiferon, mycoplasma, antinuclear antibody (ANA), anti-Smith, anti-ribonucleoprotein (RNP), anti-double-stranded DNA (dsDNA), anti-Scl-70, and anti-centromere. A chest radiograph was obtained, demonstrating mild bilateral perihilar and peribronchial thickening, as well as medial left lung base consolidation. Chest computed tomography (CT) with contrast was subsequently performed, revealing diffuse centrilobular and "tree-in-bud" nodularity throughout the lungs, most prominent in the dependent lower lobes, as well as significant distention of the esophagus with fluid and food particulate throughout its course, abruptly tapering at the GEJ (Figure 1). Given the clinical presentation and course, in tandem with the distinctive imaging findings, concern was raised for recurrent aspiration pneumonia secondary to achalasia. Additional imaging was obtained with an esophagram, showing significant esophageal dilation with limited motility, narrowing at the GEJ with "bird's beak" appearance, and minimal passage of contrast through the GEJ over time (Figure 2).

**Table 1:** Pertinent laboratory tests with results and reference ranges

Laboratory test	Results	Reference range
White blood cells	20.10 × 10^3^/uL	4.6-10.2 × 10^3^/uL
Platelets	460 × 10^3^/uL	142-424 × 10^3^/uL
Hemoglobin	10.9 g/dL	12.2-16.2 g/dL
Erythrocyte sedimentation rate	82 mm/hour	0-20 mm/hour
C-reactive protein	5.98 mg/dL	0-0.5 mg/dL
Respiratory syncytial virus	Positive	Negative
Anti-histone antibodies	Positive	Negative
Epstein-Barr virus	Negative	Negative
Cytomegalovirus	Negative	Negative
Quantiferon	Negative	Negative
Mycoplasma	Negative	Negative
Antinuclear antibody	Negative	Negative
Anti-Smith	Negative	Negative
Anti-ribonucleoprotein	Negative	Negative
Anti-double-stranded DNA	Negative	Negative
Anti-Scl-70	Negative	Negative
Anti-centromere	Negative	Negative

**Figure 3:**
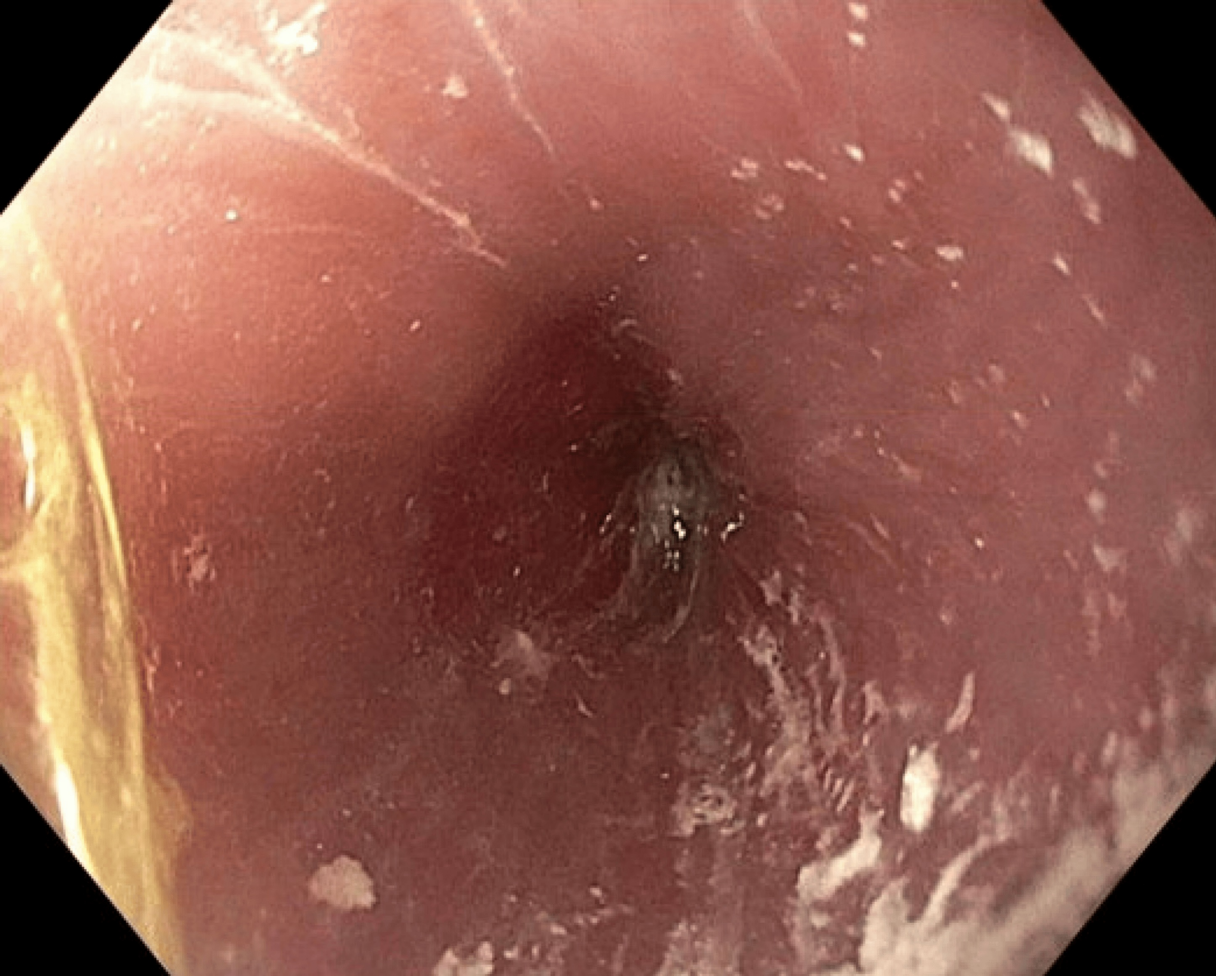
Visual inspection of the esophagus via EGD demonstrating severe narrowing at the gastroesophageal junction EGD: esophagogastroduodenoscopy

For further diagnostic evaluation and intervention, the patient underwent esophagogastroduodenoscopy (EGD) with a functional lumen imaging probe (FLIP). The EGD revealed narrowing of the esophagus (Figure 3) with severe resistance to endoscopic advancement into the stomach, with an observed distensibility of approximately 0.6 mm^2^/mmHg and a minimum diameter of 6.2 mm. FLIP topography established absent contractility using stepwise distensions, all diagnostic of achalasia, and excluded other possible etiologies such as pseudoachalasia by visual inspection. The most constricted area at the lower esophageal sphincter was successfully injected with 100 units of botulinum toxin, after which the patient experienced near-immediate relief from emesis and improved oral food tolerance the following day. Shortly after endoscopic intervention, the patient also underwent bronchoscopy with bronchoalveolar lavage to help tailor antibiotic therapy and rule out other infectious etiologies. Of note, extensive barium contrast material, from the patient's prior esophagram, was present throughout the airway, to the extent of occluding subsegmental bronchi. Relief of airway obstruction and contrast clearance were achieved with irrigation and suction.

The remainder of inpatient treatment consisted of gradual nutritional rehabilitation and completion of antibiotics for aspiration pneumonia. The patient was then discharged following evidence of clinical improvement. After three months of outpatient nutritional rehabilitation with significant improvement in growth (BMI of 18.16 kg/m^2^, 38th percentile), the patient underwent thoracoscopic esophagomyotomy for definitive management of achalasia.

## Discussion

Characterized by incomplete relaxation of the LES and ineffective or absent peristalsis of the esophageal body, achalasia represents a rare primary esophageal motility disorder manifesting as esophageal outflow obstruction and progressive dysphagia [3,4]. While the precise etiology remains elusive, current evidence suggests that an autoimmune-mediated degeneration of inhibitory neurons in the myenteric plexus, particularly those producing nitric oxide and vasoactive intestinal peptide, is involved [3-5]. Genetic predisposition and viral triggers have also been proposed in the pathogenesis of achalasia, most commonly the HLA-DQB1 allelic variant and neurotropic herpes simplex virus type 1 (HSV-1) [5]. Pediatric achalasia, representing a minority of cases, often presents with diagnostic delays due to shared symptomatology with gastroesophageal reflux disease (GERD) and carries significant nutritional and developmental implications [4,8]. Increasing use of high-resolution manometry has refined subtype classification and facilitated earlier diagnosis, which is particularly impactful in pediatric populations, where early intervention may mitigate long-term esophageal dysfunction and preserve nutritional status [9].

Therapeutic interventions for achalasia primarily focus on reducing LES pressure to facilitate esophageal emptying. First-line definitive options may include pneumatic dilation (PD), laparoscopic Heller myotomy (LHM) with partial fundoplication, and peroral endoscopic myotomy (POEM), depending on patient characteristics as well as the type of achalasia [3,4,8]. Type II achalasia has the highest response rates across all modalities, whereas type I demonstrates modest outcomes, and type III is the most difficult subtype to treat and manage [5]. As an alternative to mechanical interventions, botulinum toxin injection offers temporary relief and is typically reserved for patients who are poor surgical candidates or as a bridge to definitive surgical correction. However, additional research on the latter is needed to determine the potential impacts of botulinum toxin on surgical myotomy outcomes [4]. Oral pharmacologic agents such as nitrates or calcium channel blockers are limited by short duration and minimal effects on peristalsis and LES relaxation [3,4].

## Conclusions

Achalasia represents a rare gastrointestinal entity within the pediatric population. Our case illustrates the diagnostic and therapeutic challenges of pediatric achalasia, particularly when presenting with predominant respiratory symptoms that obscure the underlying esophageal pathology. Our patient's protracted course of presumed primary pulmonary infections underscores the potential for significant delays in diagnosis when gastrointestinal manifestations emerge later or are subtle. Through multidisciplinary collaboration and the use of complementary diagnostic modalities, a timely diagnosis can be established, and early identification and treatment can be initiated, thereby preventing long-term consequences, including adverse nutritional and developmental outcomes.
